# Pediatric oncologists' perspectives on the use of complementary medicine in pediatric cancer patients in Switzerland: A national survey‐based cross‐sectional study

**DOI:** 10.1002/cnr2.1649

**Published:** 2022-06-14

**Authors:** Léopold Pirson, Sonja C. Lüer, Manuel Diezi, Sabine Kroiss, Pierluigi Brazzola, Freimut H. Schilling, Nicolas von der Weid, Katrin Scheinemann, Jeanette Greiner, Tycho Jan Zuzak, André O. von Bueren

**Affiliations:** ^1^ Department of Pediatrics, Gynecology, and Obstetrics, Division of Pediatric Hematology and Oncology University Hospital of Geneva Geneva Switzerland; ^2^ Cansearch Research platform for pediatric oncology and hematology, Faculty of Medicine, Department of Pediatrics, Gynecology and Obstetrics University of Geneva Geneva Switzerland; ^3^ Department of Pediatrics Cliniques Universitaires Saint‐Luc Brussels Belgium; ^4^ Department of Pediatrics, Division of Pediatric Hematology and Oncology University Children's Hospital, University Cancer Center, Inselspital Bern Switzerland; ^5^ Department of Pediatrics, Division of Pediatric Hematology and Oncology Lausanne University Hospital Lausanne Switzerland; ^6^ Department of Pediatrics, Division of Pediatric Hematology and Oncology Children's Hospital Zurich Zurich Switzerland; ^7^ Department of Pediatrics Istituto Pediatrico della Svizzera Italiana Bellinzona Switzerland; ^8^ Department of Pediatrics, Division of Pediatric Hematology and Oncology Children's Hospital Lucerne Lucerne Switzerland; ^9^ Department of Pediatrics, Division of Pediatric Hematology and Oncology University Children's Hospital of Basel Basel Switzerland; ^10^ Department of Pediatrics, Division of Pediatric Hematology and Oncology Cantonal Hospital Aarau Aarau Switzerland; ^11^ Department of Pediatrics McMaster University Hamilton Hamilton Ontario Canada; ^12^ Department of Health Sciences and Medicine University of Lucerne Lucerne Switzerland; ^13^ Department of Pediatrics, Division of Pediatric Hematology and Oncology Children's Hospital of Eastern Switzerland St. Gallen Switzerland; ^14^ Department of Pediatric and Adolescent Medicine Gemeinschaftskrankenhaus Herdecke Herdecke Germany

**Keywords:** alternative medicine, cancer management, complementary medicine, pediatric cancer

## Abstract

**Background:**

There is a widespread use of complementary therapies among pediatric cancer patients. Previous studies provided evidence that communication between pediatric oncologists (POs) and patients/families about the use of these therapies is often incomplete. Furthermore, nationwide studies on this topic are rare.

**Aims:**

We assessed POs' perspectives on the use of complementary medicine (CM) in Switzerland, on the basis of an edited survey previously used in a nationwide study.

**Methods and Results:**

A link to an online survey was sent by e‐mail to each of the fifty‐two eligible pediatric oncologists in all nine Swiss Pediatric Oncology Group (SPOG) centers. Eligible respondents were board‐certified (Switzerland or abroad) POs currently working at a SPOG center. The survey was available for a total period of 2 months.

We received 29 filled questionnaires (overall response rate: 56%). Most POs (59%) indicated that they ask more than 50% of their patients about CM use. Frequent reasons for not asking about the use of CM were i) forgetting to ask (55%), ii) lack of knowledge on the subject (31%), and iii) lack of time (24%). More than every second PO (55%) reported having a lack of knowledge on the subject. A majority of POs (66% to 76%) indicated interest in learning more about specific CM topics (cannabinoids, hypnosis and relaxation, music therapy, herbal medicine, acupuncture, meditation, and yoga). More information and specific training opportunities on the use of CM was deemed important by 76% to 97% of POs.

**Conclusion:**

POs working in Switzerland identify complementary therapies as an important subject. Swiss POs are willing to acquire more knowledge on CM. More training seems to be necessary in order to increase awareness about the topic, to enhance communication about complementary therapies and thus to improve patient care.

## 1 INTRODUCTION

The term “complementary medicine” (CM) summarizes therapies which are not part of conventional medicine. Historically, the terms “complementary and alternative medicine” (CAM) were closely linked. “Alternative medicine,” referring to the use of a therapy instead of conventional medicine, is not deemed standard care and will not be discussed in this study as it seems to be inappropriate for pediatric oncologists (POs). Indeed, it should be stated that the use of additional treatment modalities should be complementary to conventional standard of care treatments, and not as an alternative to it. Thus, we will only refer to therapies used as a complement to standard of care treatments.^1^, ^2^, ^3^, ^4^

CM seem to have potential benefits, as suggested by several studies and systematic reviews.^5^, ^6^, ^7^, ^8^, ^9^, ^10^, ^11^, ^12^, ^13^, ^14^ The inclusion of CM in conventional medicine is a way to offer a more holistic approach to the patient and the family, which leads to the concept of “integrative medicine,” which has been used more and more as it better corresponds to POs' current medical practice. Integrative medicine does not only include physical aspects, but also psychic and spiritual aspects of the human being, regarding them in a holistic way.^1^, ^15^

CM can be subdivided in four main groups: biochemical therapies (e.g., aromatherapy, dietary complements including antioxidants and vitamins), bioenergetic therapies (e.g., anthroposophic medicine, homeopathy), biomechanical therapies (e.g., chiropractic) and mind‐body based therapies (e.g., hypnosis, music therapy).^1^

Previous studies (USA,^16^ UK,^17^ Germany,^18^ and Turkey^19^) and systematic reviews^20^ have emphasized the widespread use of CM by pediatric cancer patients. Worldwide, the prevalence of any CM use in children with cancer (since cancer diagnosis) varies massively, ranging from 6% to 91% in a systematic review.^21^ A study showed that although the use of CM appears more frequent in lower income countries with an average prevalence of approximately 77%, higher income countries also showed an important frequency of CM use (average prevalence of approximatively 47%).^22^

CM are mostly used by the pediatric cancer patients as a way to increase wellness, but also to ease the symptoms related to chemotherapies, to reinforce the immune system and to improve healing.^15^, ^23^, ^24^ Some CM modalities are widely used in conventional practice, especially in the management of procedural pain (by repeated venous, port, lumbar and bone marrow punctures) or stress and anxiety generated by the side effects of chemotherapies (e.g., hypnosis, music therapy, acupuncture, aromatherapy and others).^15^, ^25^, ^26^, ^27^, ^28^

In Switzerland, there has been an increasing interest in complementary therapies. A recent study showed that 97% of all pediatricians in Switzerland were asked by their patients about the use of CM, and two thirds of them were interested in further information and training about complementary and integrative medicine (CIM).^29^ Today, there is an official recognition for homeopathy, anthroposophic medicine, traditional Chinese medicine, acupuncture, neural therapy and phytotherapy, with structured and continuous postgraduate formation programs.^30^ Officially mandated by the Swiss Society of Pediatrics (SSP), the Swiss Interest Group for Integrative Pediatrics (SIGIP) was founded in 2017 with the aim to create a national platform of pediatricians interested in complementary therapies, providing an important expertise on the subject and organizing trainings.^30^

The experience of colleagues at the University of Bern showed that 53% of their pediatric cancer patients were using CM. The oncologist was not aware of this use in approximately ¼ of cases, and half of the families were expecting more information about CM.^23^ More recently, the pediatric oncology team of the University of Lausanne identified a higher use of CM after diagnosis (69.3%) than before diagnosis (54.3%) among their patients, with a marked increase of use of hypnosis during oncologic treatment, likely due to local practice of the medical team to cope with procedural pain. There appears to be a need to improve communication, as only two thirds of patients/parents inform their oncologist about CM use.^31^

Internationally, the perspective of POs with regards of CM use of their patients has been studied in a few studies. In the United States, more than 50% of the interviewed POs thought that dietary supplements, herbal medicine, special diets, vitamins, and chiropractic therapy might be harmful to patients.^2^ A German study reported that half of the interviewed POs were unable to acquire CM knowledge during medical training and over 70% of them suggested that CM should be an integral part of postgraduate training.^32^ A more recent German study highlighted an important need for more information about CM by POs.^1^

CM use among pediatric oncology patients in Switzerland has been already investigated and the study revealed an important need for further communication with their POs.^31^ There is no study investigating Swiss POs views on their patients' use of CM. The aim of this study is to explore POs' perception of (1) the use of CM among their patients, (2) the communication about CM with their patients, (3) their collaboration with CM specialists/therapists, and (4) their need for further learning on the subject. Furthermore, this study may increase the awareness for this topic and may consecutively stimulate the communication about CM between pediatric cancer patients/families and their physicians, improving pediatric oncology patients' management.

## 2 METHODS

### 2.1 Subjects and eligibility criteria

A link to an online survey was sent by e‐mail to each local investigator—participating in the design of this cross‐sectional study—from all nine Swiss Pediatric Oncology Group (SPOG) centers (Aarau, Basel, Bellinzona, Bern, Geneva, Lucerne, St Gallen, Lausanne, and Zurich). Each local investigator forwarded it to their eligible local pediatric oncology colleagues and collected the number of potential responses in their center. The survey was available for a total period of 2 months (from 17 June through 17 August 2021). Reminders to the local investigator were sent 1 month after initial survey distribution. The data were anonymous and we did not collect any participant's identifying data.

All answered forms were anonymous and were only accessible to the main author. The e‐mails were exclusively exchanged through professional e‐mail addresses which are secured by each hospital's network security system (in general by the HIN security network of Switzerland, providing the best online security in Switzerland). The study data was collected through a survey created using Google Forms, which is Google's online form and survey program with a high level of data security. After publication, copies of the data will be stored on a password‐protected institutional computer and the survey will be deleted.

Eligibility criteria to answer the survey were board‐certified (Switzerland or abroad) pediatric oncologists currently working in a SPOG center either in clinical care program for pediatric oncology patients aged 0 to 18 years, or in a research program or other not directly patient related work. There were no exclusion criteria. A total of fifty‐two POs were eligible and received the survey, and twenty‐nine of them responded the survey.

### 2.2 Questionnaire

Roth's questionnaire^2^ was adapted to local practice and changes ‐ mainly with the aim to increase the clarity of the survey ‐ were made based on our local investigators' suggestions. The survey consisted of 27 questions (Supplementary File).

First, POs were asked about personal information such as gender, graduation in or outside of Switzerland, number of years of pediatric oncology practice, area of practice, allocated time to clinical and non‐clinical practice, and acquired qualifications related to CM.

Then they were asked about the use of CM among their patients and their interactions with them concerning CM, as for example what percentage of patients is using any kind of CM, how often the oncologist asks the patients if they are using CM, reasons why not asking for it, how often does the patient ask spontaneously for CM, why POs are not comfortable discussing CM and how do they react when the patient addresses the topic. Also, they were asked about their need for information and about the availability of resources as well as experts concerning CM (pharmacist who assesses potential interactions, CM therapist), and how often do they have information exchange with CM specialists concerning patients currently using CM. Questions were included about their perception of CM therapy such as potential benefits and harms for every kind of CM, and their need of more information and training on every type of CM.

### 2.3 Statistical analysis

Descriptive statistics were generated for all variables. Subgroup analyses were performed with Fisher exact tests to evaluate the relationships among physicians' demographic characteristics and the following variables: communication with the patient/family, referral to a CM specialist/provider, need to do literature search to get information about CM. Statistical analysis and graphs were performed using the software GraphPad Prism 9.2.0 (GraphPad Software Inc., San Diego, California, US). No assessment of risk of bias was performed.

## 3 RESULTS

### 3.1 Study population

The questionnaire was sent to 52 POs working in Switzerland who confirmed being eligible for the study. We received 29 filled questionnaires (overall response rate 56%). Response rate in all three parts of Switzerland were 16/18 (89%), 12/33 (36%) and 1/1 (100%) in French, German and Italian‐speaking part of Switzerland, respectively. All questionnaires were fully completed, and none was excluded. Study population was assessed by a series of demographic questions. Respondents' demographic data are reported in Table 1.

**TABLE 1:** Characteristics of responding persons

Characteristics	*n* = 29 (%)
Sex	
Female	17 (59)
Male	12 (41)
Number of years in practice in pediatric oncology	
5–10 years	5 (17)
10–20 years	18 (62)
>20 years	6 (21)
Medical school attended	
Switzerland	16 (55)
Outside of Switzerland	13 (45)
Switzerland region of practice	
German speaking	12 (42)
French speaking	16 (55)
Italian speaking	1 (3)
Clinical care allocated time	
≥80%	16 (55)
≥50%–80%	9 (31)
<50%	4 (14)
CM qualifications	
Hypnosis	1 (3)
None	28 (97)

### 3.2 Communication of pediatric oncologists with the patients/families regarding CM

We analyzed communication between POs and their patients about CM (Table 2). Most POs (59%) do ask to more than half of their patients about CM in general, and particularly about biochemical therapies such as dietary supplements and special diets (55%), and mind‐body based therapies such as hypnosis, meditation, and music therapy (38%). POs ask less frequently about the use of other subgroups of CM. Twenty‐one percent of POs ask less than 25% of their patients about the use of CM. The main reasons why POs do not ask their patients about CM are forgetting to ask (55%), lack of knowledge on the subject (31%) and lack of time (24%), as illustrated in Supplementary Table 1.

**TABLE 2:** Percentage of POs asking patients about the use of CM in general and subgroups of CM (*n* = 29)

	Ask none of patients	Ask 1%–10% of patients	Ask 11%–25% of patients	Ask 26%–50% of patients	Ask 51%–75% of patients	Ask >75% of patients
All CM considered	3.5% (1)	10.3% (3)	6.9% (2)	20.7% (6)	17.2% (5)	41.4% (12)
Biochemical therapies	6.9% (2)	6.9% (2)	6.9% (2)	24.1% (7)	6.9% (2)	48.3% (14)
Bioenergetic therapies	20.8% (6)	17.2% (5)	6.9% (2)	17.2% (5)	6.9% (2)	31% (9)
Biomechanical therapies	31% (9)	27.6% (8)	6.9% (2)	6.9% (2)	3.5% (1)	24.1% (7)
Mind‐body based therapies	20.7% (6)	10.3% (3)	20.7% (6)	10.3% (3)	10.3% (3)	27.7% (8)

**FIGURE 1:**
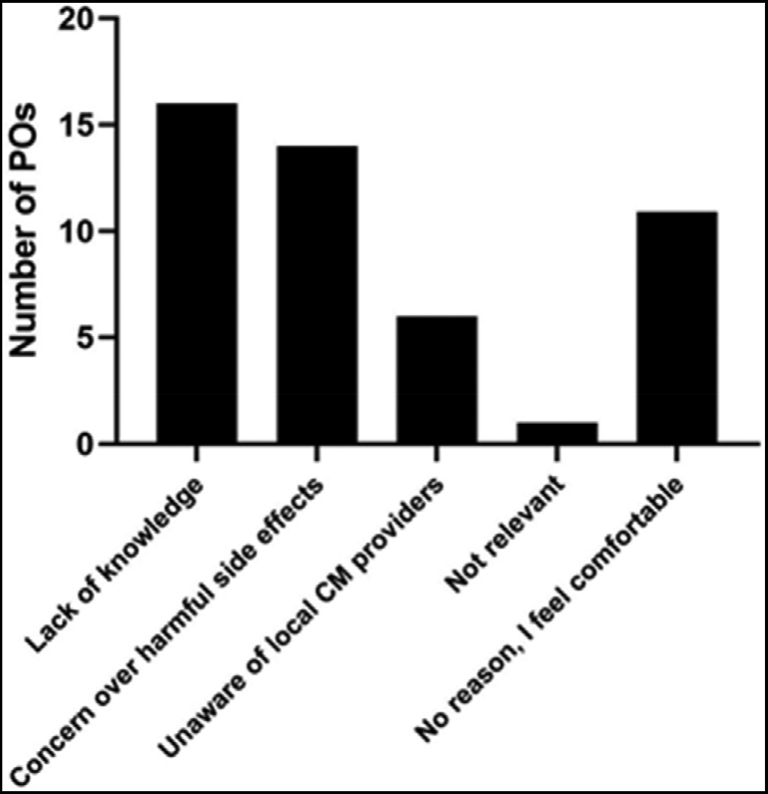
Reasons why Pediatric Oncologists in Switzerland do not feel comfortable to discuss CM with their patients and families (more than one answer possible; “lack of knowledge”: 16/29; “concern over harmful side effects”: 14/29; “unaware of local CM providers”: 6/29; “not relevant”: 1/29, “no reason, I feel comfortable”: 11/29)

More than half of POs (55%) reported feeling uncomfortable discussing CM therapies because of a lack of knowledge and education on the topic, and almost half of POs (48%) reported that they had concern about potential harmful side effects of CM. Fewer (17%) responded that they were unaware of local providers. Less than half of POs (38%) reported feeling completely comfortable talking about CM (Figure 1).

Most respondents (83% to 97%) reported that less than 50% of their patients and families had initiated a conversation about the different subgroups of CM. The main reaction of POs uncomfortable when asked about CM by their patients and families were to admit that they were not knowledgeable on the subject (62%) and referring them to a CM specialist or asking a pharmacist (59%), as shown in Supplementary Table 2.

Overall, all but one POs are open to discuss about CM with patients with a good prognosis and all of POs are open to discuss the subject with patients with poor prognosis.

### 3.3 Physicians estimates about the use of CM

We analyzed the estimates of POs about use of CM among their patients/families (Table 3). For all subgroups of CM, most of the POs estimated that up to 75% of their patients were using CM on a regular basis. Sixty‐nine percent of POs estimated that at least 10% of their patients used biochemical therapies (e.g., aromatherapy, antioxidants, dietary complements, melatonin) on a regular basis, 62% of POs believed that more than 10% of their patients regularly used bioenergetic therapies (e.g., acupuncture, anthroposophic medicine, homeopathy) and mind‐body based therapies (e.g., hypnosis, meditation, music therapy).

**TABLE 3:** POs estimates about the use of CM of their patients (*n* = 29)

	None of patients uses CM	1%–10% of patients use CM	11%–25% of patients use CM	26%–50% of patients use CM	51%–75% of patients use CM	>75% of patients use CM	Don't know
Biochemical therapies	3.5% (1)	27.6% (8)	31% (9)	10.3% (3)	24.1% (7)	3.5% (1)	0% (0)
Bioenergetic therapies	0% (0)	34.5% (10)	24.1% (7)	17.2% (5)	13.8% (4)	6.9% (2)	3.5% (1)
Biomechanical therapies	10.3% (3)	45% (13)	20.7% (6)	13.8% (4)	3.4% (1)	3.4% (1)	3.4% (1)
Mind‐body based therapies	6.9% (2)	27.6% (8)	20.7% (6)	27.6% (8)	3.4% (1)	10.3% (3)	3.5% (1)

### 3.4 Referral to a CM specialist/therapist

**FIGURE 2:**
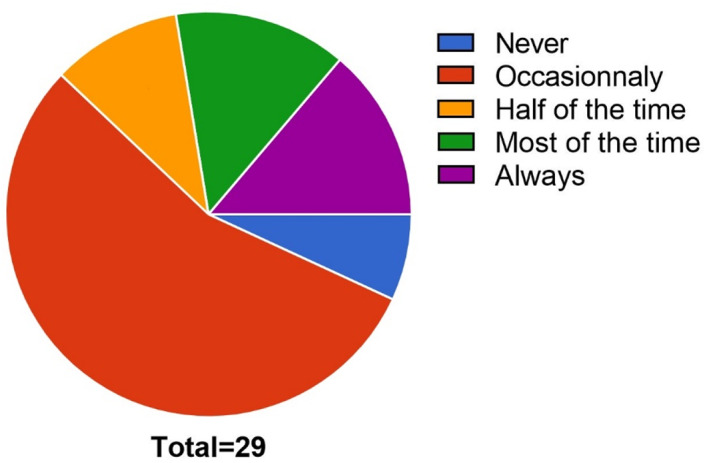
Patient referral frequencies to CM specialists according to the practice of individual Pediatric Oncologist in Switzerland (“never”: 2/29; “occasionally”: 16/29; “half of the time”: 3/29; “most of the time”: 4/29; “always”: 4/29)

We investigated referral rates of patients to a CM specialist/therapist. Referral rates appears to be relatively low, as most POs refer occasionally their patients to a CM specialist/therapist (16/29; 55%—Figure 2).

Patients referred to a CM specialist by POs are mainly referred to hypnotherapist and massage therapist (69%), followed by homeopathic practitioner (55%), osteopath and anthroposophic therapist (52%), and acupuncturist (48%). Most of the time, the patient is referred to a CM specialist on demand of the family (63%–79%). Patients known to be interested in CM are not referred in approximately one third of cases for massage therapy and hypnosis (31%), half of cases for homeopathy (45%), anthroposophic medicine and osteopathy (48%), acupuncture (52%) and in lesser cases for chiropractic and dietary specialist (59%) and yoga (72%). Referral rates of patients to each CM specialists are detailed in Supplementary Figure 1.

Most respondents (90%) do have a pharmacist who is able to assess potential medical interactions with CM treatment. More than two third (66%–93% depending on the type of CM) of POs do not have a CM specialist available in their network for the following therapies: aromatherapy, antioxidant, black seed oil, curcuma, enzymes, pre and probiotics, Ayurveda, magnets, Reiki, cranio‐sacral therapy, guided imagery, horse‐riding therapy, and martial arts. Nevertheless, more than half of POs (52%–83% depending on the type of CM) do have an in‐house or external specialist for the following therapies: hypnosis, music therapy, massage therapy, melatonin, cannabinoids, homeopathy special diet, and acupuncture. Details are shown in Supplementary Table 3. More than half of POs (52%) consider their communication with CM specialist as good.

### 3.5 Estimations of risks and benefits of CM by the physicians

POs' estimations of risks and benefits of CM are shown in Table 4. POs consider that CM may be efficient in improving the quality of life or specific symptoms for pediatric oncology patients in a curative treatment setting, as for cannabinoids/music therapy/relaxation (93%), massage therapy (90%), hypnosis/meditation (79%), and melatonin (76%). There is no clear overall certainty of ineffective CM, but there is a mixed opinion for all the other CM, with a more evident uncertainty about some CM such as antioxidants, black seed oil, curcuma, enzymes, Ayurveda, Reiki, cranio‐sacral therapy, and guided imagery (>50% of “don't know” responses).

**TABLE 4:** POs perspective about the risks and benefits and further learning of different types of CM (*n* = 29)

Therapy	May be effective	May be harmful	Interest in further learning
**Biochemical**			
Aromatherapy	45% (13)	21% (6)	45% (13)
Antioxidants	17% (5)	34% (10)	41% (12)
Black seed oil	7% (2)	17% (5)	31% (9)
Cannabinoids	93% (27)	24% (7)	76% (22)
Curcuma	28% (8)	17% (5)	55% (16)
Dietary Supplement	45% (13)	45% (13)	55% (16)
Enzymes	7% (2)	41% (12)	28% (8)
Herbal Medicine	59% (17)	59% (17)	66% (19)
Melatonin	76% (22)	7% (2)	55% (16)
Mistletoe Therapy	41% (12)	21% (6)	52% (15)
Pre and Probiotics	34% (10)	45% (13)	52% (15)
Special Diet	34% (10)	45% (13)	55% (16)
Vitamins	45% (13)	34% (10)	52% (15)
**Bioenergetics**			
Acupuncture	59% (17)	24% (7)	66% (19)
Anthroposophic medicine	34% (10)	21% (6)	59% (17)
Ayurveda	24% (7)	10% (3)	41% (12)
Homeopathy	52% (15)	21% (6)	55% (16)
Magnets	10% (3)	14% (4)	21% (6)
Reiki	17% (5)	7% (2)	34% (10)
**Biomechanical**			
Chiropractic	34% (10)	31% (9)	38% (11)
Cranio‐sacral Therapy	21% (6)	24% (7)	38% (11)
Massage Therapy	90% (26)	17% (5)	59% (17)
**Mind‐body**			
Guided Imagery	28% (8)	7% (2)	52% (15)
Horse Riding Therapy	69% (20)	24% (7)	41% (12)
Hypnosis	79% (23)	7% (2)	76% (22)
Martial Arts	52% (15)	31% (9)	52% (15)
Meditation	79% (23)	3% (1)	66% (19)
Music Therapy	93% (27)	3% (1)	69% (20)
Relaxation	93% (27)	3% (1)	76% (22)
Yoga	72% (21)	7% (2)	62% (18)

Although most of the CM do not seem harmful for more than half of respondents such as all mind‐body based therapies (41%–93%; average 77%), massage therapy (76%), melatonin (69%), homeopathy (66%), aromatherapy/cannabinoids (59%), herbal medicine is considered to be potentially harmful to the patients for 59% of respondents. There is also an important uncertainty about potential harmful effects of some CM such as black seed oil, Ayurveda, Reiki, and guided imagery (>50% of “don't know” responses).

All POs indicated that it is important for them to know about CM therapies their patients are using, in order to prevent potential harmful drug interactions (100%), improve trust between physicians and patients, improve patient adherence to medical therapy (86%) or improve patient satisfaction with their medical therapy (79%).

### 3.6 Interest of POs for further information and training

Interest for more information and training on CM was assessed. It appears that many POs (72%) do some literature searches to get information about side effects and interaction related with CM at least from time to time and nearly half of POs do it often/very often (45% of POs).

Most POs (66% to 76%) are interested in learning more about the following CM: cannabinoids, hypnosis and relaxation, music therapy, herbal medicine, acupuncture, meditation, and yoga. Many POs (62% to 79%) are not interested in further information about the following CM: magnets, enzymes, black seed oil, Reiki, chiropractic and cranio‐sacral therapy. There is, in general, more interest in mind‐body therapies (41%–76%; average 62%) than in other subgroups of CM (21%–76%; average 49%) (Table 4).

**FIGURE 3:**
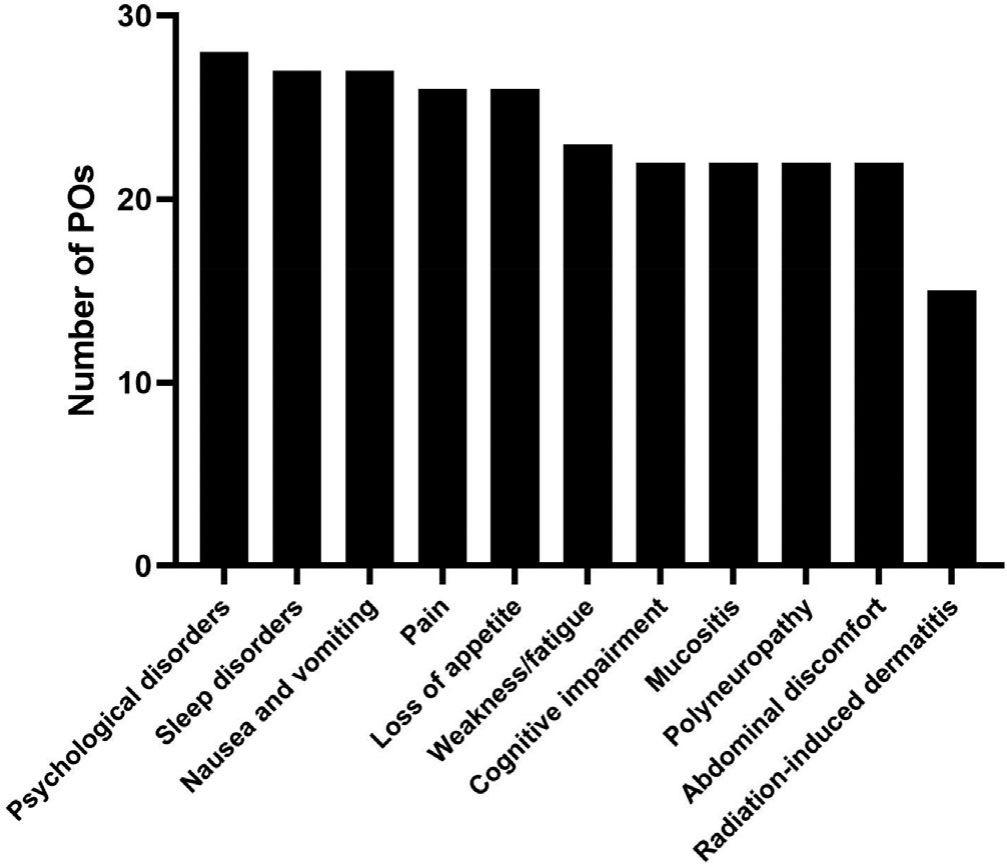
Number of POs considering further training with regards of the use of CM for specific symptoms and side‐effects as important (multiple answers possible)

As shown in Figure 3, a large majority of POs (76%–97%; average 84%) respond that information or training opportunities on the use of CM for treating symptoms of cancer or side effects of anti‐tumoral therapy in pediatric oncology patients would be important for their clinical work, except for radiation‐induced dermatitis (52%). More than half of POs (52%–59%) think it would be very important for specific symptoms such as nausea and vomiting, pain, loss of appetite, changes in taste, weakness, sleep disorders, and psychological disorders (Figure 3; Supplementary Table 4).

### 3.7 Subgroup analysis

Subgroup analysis did not show any difference between physicians' demographic characteristics and the following variables: communication with the patient/family, referral to a CM specialist/provider, need to do literature research to get information about CM (data not shown).

## 4 DISCUSSION

In Switzerland, CM is often used and considered as an important subject by POs.^15^, ^23^, ^29^, ^30^, ^31^ Our survey shows that POs in Switzerland are generally aware that many of their cancer patients use CM regularly and that they are concerned about potential harmful side effects of CM. All of them indicated that it is important to know CM therapies their patients are using.

The communication of POs with their patients and families about CM seems to be incomplete, as the topic is not addressed systematically by all POs. Indeed, 59% of POs do ask to more than half of their patients. This rate is comparable to the frequency described in Roth's study (50%) for POs in the US.^2^ The reasons why Swiss POs do not ask all the time are mostly related to a prioritization of their patients' problems as they often forget or don't have enough time in their schedule to discuss the topic, but also seem to be related to a lack of knowledge on the subject.

The topic is relatively infrequently actively discussed by the patients and families as reported by most POs (less than 50% of patients according to 83% to 97% of respondents), probably because they think that their PO may be not knowledgeable on the subject, or because they might fear their PO's reaction. Indeed, POs' most frequent answer to patients asking about CM is not being knowledgeable on the subject (62%). Only few of them seem to have a negative perception by convincing the patient not to use CM (14%). In a recent Swiss study focusing on the CM use in pediatric oncology patients, only 38% of all respondents stated that they have discussed CM with their POs, and that the discussion was initiated by one of their parents in 87% of cases, which is in disagreement with POs perspective described in our study. These observations highlight a desire for more communication between patients and POs about CM. The same study reported a substantial concern about a negative reaction from POs, preventing some patients to discuss about CM with them.^31^

In our study, POs are generally open to discuss CM with both good and poor prognosis pediatric oncology patients. Based on our data, we are not able to evaluate the effect of CM on prognosis. However, we assume that discussing about CM could improve the patient's well‐being, allow the family to support the child in an active and medically safe manner and enhance compliance to the conventional therapy. In order to evaluate the effect of CM on the prognosis and outcome of pediatric oncology patients undergoing CM in addition to conventional treatment versus conventional treatment alone, further studies should be performed.

Very few POs in Switzerland are trained for CIM, as only one respondent of our study has an additional CIM‐related qualification (hypnosis). POs are aware of their lack of knowledge and training on CIM. One third of them indicated that their lack of knowledge prevents them from asking their patients about the use of CM, and more than half of them are uncomfortable talking about CM with their patients and families.

Several POs consider that some CM ‐ including music therapy, relaxation, massage therapy, hypnosis, meditation, cannabinoids and melatonin ‐ could improve the quality of life and specific symptoms for their patients. There is an important uncertainty among POs on potential risks or benefits of specific CM.

This leads to an important need for further learning and training about CIM, especially for mind‐body therapies, cannabinoids, herbal medicine and acupuncture (66% to 76% of POs), for their potential to ease treatment‐ or disease‐induced complaints. The most important application areas of CIM for POs appears to be nausea/vomiting, lack of appetite, pain, fatigue, sleep disorders and psychological disorders. This is in agreement with findings of a previous study performed in Germany.^1^

In Switzerland, there is no systematic CIM training program during POs' formation. This highlights the importance of the Swiss Interest Group for Integrative Pediatrics (SIGIP), whose members offer training programs on CIM.^30^

Our survey shows that the collaboration with CIM specialist is not yet very well established. This is surprising, because POs' lack of knowledge on CIM should lead to a high referral rate to CIM specialists/therapist but paradoxically, referral rate is low with almost two third of POs occasionally or never referring their patients. The main reasons for this observation appear to be the availability of a pharmacist in the network assessing for medical interaction as well as the lack of CIM specialists in their network.

This study is potentially limited by a demographic respondent bias. Despite a relatively good overall response rate (56%), there is a lower response rate in the German‐speaking part of Switzerland (36%). Furthermore, it is likely that POs who responded to the survey were more interested by CIM than non‐respondents, with a higher interest to learn more about CIM. This hypothesis is supported by a recent paper investigating the attitudes of healthcare coworkers towards CM in Turkey. The cross‐sectional study using a survey showed an impressive response rate (83%) with 794 healthcare coworkers completing the survey. Of interest was the more negative attitude towards CM of physicians when compared to other healthcare professions.^33^

The risk for bias was not assessed although it does exist, as we received only approximatively half of the potential answers. As the survey was responded in an anonymous manner, we are unable to compare the information of respondents (*n* = 29) and non‐respondents (*n* = 23). In addition, we emphasize that this study is also limited to a small group of participants and the results of this pilot observation should be treated with caution.

In summary, there is a need to increase communication and interaction between patients/families and POs with regards to CM. It appears to be reasonable to implement a systematic CIM training program for POs. This may improve care provided to pediatric cancer patients in Switzerland by offering them a more holistic and individual approach of care, by limiting potential harms caused by an inappropriate use of CM, and by improving the trust‐based relationship between the medical team/physician and the family/patient.

## AUTHOR CONTRIBUTIONS

**Léopold Pirson:** Investigation (equal); writing – original draft (equal). **Sonja Lüer:** Investigation (supporting); writing – review and editing (supporting). **Manuel Diezi:** Investigation (supporting); writing – review and editing (supporting). **Sabine Kroiss:** Investigation (supporting); writing – review and editing (supporting). **Pierluigi Brazzola:** Investigation (supporting); writing – review and editing (supporting). **Freimut Schilling:** Investigation (supporting); writing – review and editing (supporting). **Nicolas von der Weid:** Investigation (supporting); writing – review and editing (supporting). **Katrin Scheinemann:** Investigation (supporting); writing – review and editing (supporting). **Jeanette Greiner:** Investigation (supporting); writing – review and editing (supporting). **Tycho Jan Zuzak:** Writing – original draft (equal); writing – review and editing (equal). **Andre von Bueren:** Investigation (lead); supervision (lead); writing – original draft (equal).

## CONFLICT OF INTEREST

The authors have stated explicitly that there are no conflicts of interest in connection with this article.

## ETHICS STATEMENT

This study was considered as falling outside of the scope of the Swiss legislation regulating research on human subjects, so that the need for local ethics committee approval was waived (confirmed by the local ethics committee; Req‐2021‐01340). Completion of the electronic survey was viewed as consent to participate and to use the anonymous responses in our analysis and publications.

## Supporting information

Supplementary File. Survey consisting of 27 questions used for the study
**Supplementary Table 1**. Reasons (more than one answer possible) POs do not ask their patients about CM (*n* = 29).
**Supplementary Table 2**. Response (more than one answer possible) to the patients asking about CM that PO is not comfortable discussing (*n* = 29).
**Supplementary Table 3**. Percentage of POs having access to an available CM specialist in their network (*n* = 29).
**Supplementary Table 4**. Importance for practice of information and training on the use of CM for specific symptoms or side‐effects (*n* = 29).

**Supplementary Figure 1**. Number of POs referring to specific CM providers.

## Data Availability

The data that support the findings of this study are available from the corresponding author upon reasonable request.
