# Supplementary material for: Pediatric oncologists' perspectives on the use of complementary medicine in pediatric cancer patients in Switzerland: A national survey‐based cross‐sectional study
Source: Cancer Rep (Hoboken). 2022 Jun 14;6(1):e1649. doi: 10.1002/cnr2.1649 (PMC9875643; doi:10.1002/cnr2.1649)
Supplement: Supplementary file 1 — Supplementary File. Survey consisting of 27 questions used for the study Supplementary Table 1. Reasons (more than one answer possible) POs do not ask their patients about CM (n = 29). Supplementary Table 2. Response (more than one answer possible) to the patients asking about CM that PO is not comfortable discussing (n = 29). Supplementary Table 3. Percentage of POs having access to an available CM specialist in their network (n = 29). Supplementary Table 4. Importance for practice of information and training on the use of CM for specific symptoms or side‐effects (n = 29). [file CNR2-6-e1649-s001.docx]

SUPPLEMENTARY TABLE 1. Reasons (more than one answer possible) POs do not ask their patients about CM (n=29).

|  | **Percentage of POs** |
| --- | --- |
| Forget to ask | 55.2% (16) |
| Lack of knowledge on the subject | 31% (9) |
| Lack of time | 24.1% (7) |
| Don’t think it’s important | 13.8% (4) |
| Uncomfortable discussing the topic | 3.4% (1) |
| Does not apply, always ask | 27.6% (8) |

SUPPLEMENTARY TABLE 2. Response (more than one answer possible) to the patients asking about CM that PO is not comfortable discussing (n=29).

|  | Percentage of POs |
| --- | --- |
| Tell not being knowledgeable on the therapy | 62% (18) |
| Refer them to a CM specialist | 59% (17) |
| Give them information from internet or pamphlets | 17% (5) |
| Tell not to use the therapy | 14% (4) |
| Does not apply, always comfortable discussing this | 28% (8) |

SUPPLEMENTARY TABLE 3. Percentage of POs having access to an available CM specialist in their network (n=29).

| Therapy | In-house | External |
| --- | --- | --- |
| **Biochemical** |  |  |
| Aromatherapy | 17% (5) | 14% (4) |
| Antioxidants | 7% (2) | 7% (2) |
| Black seed oil | 3% (1) | 3% (1) |
| Cannabinoids | 52% (15) | 10% (3) |
| Curcuma | 14% (4) | 10% (3) |
| Dietary Supplement | 38% (11) | 10% (3) |
| Enzymes | 3% (1) | 10% (3) |
| Herbal Medicine | 14% (4) | 31% (9) |
| Melatonin | 59% (17) | 7% (2) |
| Mistletoe Therapy | 7% (2) | 41% (12) |
| Pre and Probiotics | 24% (7) | 7% (2) |
| Special Diet | 45% (13) | 7% (2) |
| Vitamins | 28% (8) | 10% (3) |
| **Bioenergetics** |  |  |
| Acupuncture | 21% (6) | 31% (9) |
| Anthroposophic medicine | 3% (1) | 41% (12) |
| Ayurveda | 7% (2) | 14% (4) |
| Homeopathy | 21% (6) | 38% (11) |
| Magnets | 0% (0) | 7% (2) |
| Reiki | 3% (1) | 7% (2) |
| **Biomechanical** |  |  |
| Chiropractic | 7% (2) | 31% (9) |
| Cranio-sacral Therapy | 10% (3) | 17% (5) |
| Massage Therapy | 41% (12) | 24% (7) |
| **Mind-body** |  |  |
| Guided Imagery | 14% (4) | 10% (3) |
| Horse Riding Therapy | 0% (0) | 34% (10) |
| Hypnosis | 59% (17) | 24% (7) |
| Martial Arts | 7% (2) | 14% (4) |
| Meditation | 21% (6) | 28% (8) |
| Music Therapy | 69% (20) | 10% (3) |
| Relaxation | 24% (7) | 24% (7) |
| Yoga | 7% (2) | 31% (9) |

SUPPLEMENTARY TABLE 4. Importance for practice of information and training on the use of CM for specific symptoms or side-effects (n=29).

| Symptom/side-effect | Very important | Rather important | Rather not important | Not important at all |
| --- | --- | --- | --- | --- |
| Psychological disorders | 59% | 38% | 3% | 0% |
| Sleep disorders | 59% | 34% | 7% | 0% |
| Pain | 59% | 31% | 10% | 0% |
| Nausea and vomiting | 52% | 41% | 7% | 0% |
| Loss of appetite, changes in taste | 52% | 38% | 10% | 0% |
| Tiredness/weakness/fatigue | 52% | 27% | 21% | 0% |
| Cognitive impairment | 45% | 31% | 21% | 3% |
| Mucositis | 41% | 35% | 17% | 7% |
| Polyneuropathy | 38% | 38% | 21% | 3% |
| Abdominal discomfort | 35% | 41% | 24% | 0% |
| Radiation-induced dermatitis* | 35% | 17% | 38% | 7% |

* One respondent answered “no answer possible”
