# Supplementary figures and images for: Pediatric oncologists' perspectives on the use of complementary medicine in pediatric cancer patients in Switzerland: A national survey‐based cross‐sectional study
Source: Cancer Rep (Hoboken). 2022 Jun 14;6(1):e1649. doi: 10.1002/cnr2.1649 (PMC9875643; doi:10.1002/cnr2.1649)

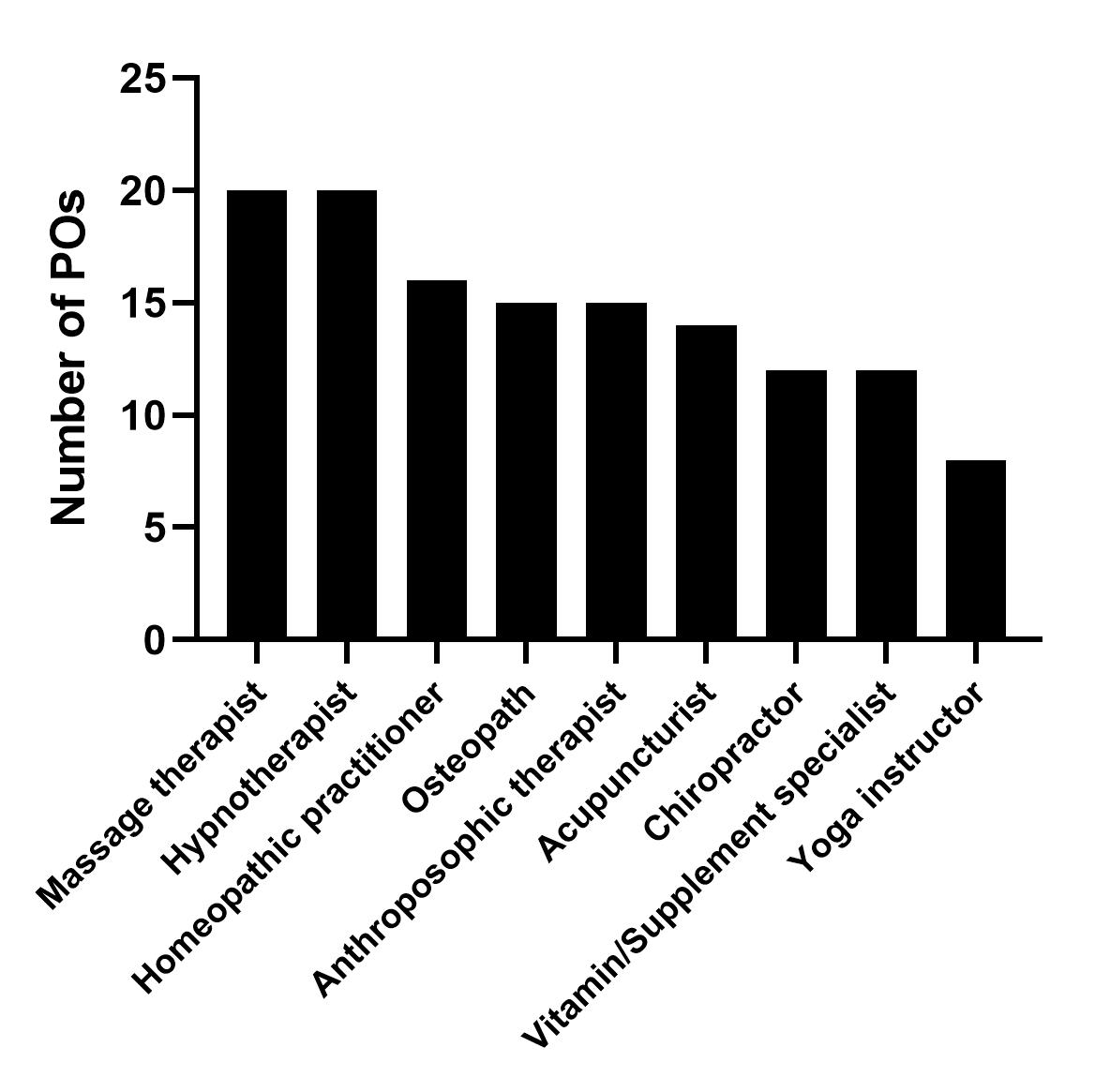


SUPPLEMENTARY FIGURE 1: Number of POs referring to specific CM providers.

Supplement: Supplementary file 2 — Supplementary Figure 1. Number of POs referring to specific CM providers. [file CNR2-6-e1649-s002.docx]
